# Occupational health and safety management when digitalizing public services: Local government manager experiences in Sweden

**DOI:** 10.1177/10519815251403559

**Published:** 2026-02-03

**Authors:** Annica Asp, Kristina Palm, Carin Håkansta

**Affiliations:** 1 Department of Working Life Science, Karlstad University, Karlstad, Sweden; 2 Unit of Occupational Medicine, Karolinska Institutet, Stockholm, Sweden

**Keywords:** occupational health, occupational injury prevention, organizational design and management, psychological and social factors at the workplace (psycho­social work environ­ment factors), technology, workplace health promotion

## Abstract

**Background:**

Strained financial resources and increasing demands for high-quality services in an ageing population are two challenges facing local government managers. While digitali­zing public service processes can meet these challenges, Occupational Health and Safety Manage­ment (OHSM) is often overlooked.

**Objective:**

This study explores how Swedish local government managers handle digitalization processes in relation to OHSM, identifying the potential drivers and barriers that influence their integration.

**Methods:**

The study is based on 25 semi-structured interviews with managers, HR profes­sionals, strategists, and Health and Safety Representatives (HSRs) in two Swedish muni­cipa­lities. An inductive approach, inspired by the Gioia methodology, was used. The Plan-Do-Check-Act (PDCA) model was utilized to analyze where potential drivers for and barriers to integrate digitalization into OHSM emerge.

**Results:**

School managers demonstrated a systematic approach to integrating digitalization with OHSM, collaborating with stakeholders and managing procurement despite financial con­straints. In contrast, social and home care managers reflected technological determinism, overlooking risk assess­­ments and Occupational Health and Safety (OHS) evaluations, thus under­­estimating OHS risks and limiting the involvement of employees and HSRs.

**Conclusions:**

The study demonstrates that managerial approach and organizational context are crucial for integrating OHS into digitalization processes. By highlighting the value of the PDCA model as a guiding framework, it advances the under­standing of OHSM as a dynamic, context-sensitive process with practical relevance for management in technology-driven environ­ments.

## Introduction

Public sector managers face a wide range of challenges, including demographic shifts, econo­mic recession, the aftermath of the COVID-19 pandemic, and an ageing workforce.^1^ In addition, growing demands to adopt advanced digital techno­logies,^2^ such as automated-decision processes,^3^ further comp­licates the managerial landscape. In Sweden, local govern­ment managers are encou­raged to implement digital technologies wherever possible, aligning with the national ambition to become a global leader in digitalization.^4^ These implemen­tations aim to enhance efficiency, transparency and accountability within the public sector.^5^ However, digital work environments may also introduce challen­ges that negatively affect employee well-being.^6–9^

In 2023, a Swedish labor inspection campaign focusing on digitalization found that 23 percent of managers failed to meet their Occupational Health and Safety Management (OHSM) respon­sibilities.^10^ This finding underscores the need for greater knowledge of how digital techno­logy can be integrated in ways that support a healthy work environment. This article builds on existing practices and research related to OHSM, which refers to the systematic mana­ge­ment of safety and health within organizations, encompassing policies, systems, records and stan­dards.^11,12^ The article focuses on OHSM´s core objective: to safeguard employees’ health, safety, and welfare by promoting a supportive work environment.^12,13^ An effective OHSM system enhances the identification of preventive measures through continuous evaluations that enable timely and appropriate improvements.^11,14^ Although research on OHSM has expanded since 2000,^15,16^ few studies have addressed the specific challenges posed by digitali­zation.^6,17^

Despite a long-standing tradition of OHSM in Sweden, the number of published articles on the topic remains limited.^16^ This scarcity of research is concerning, given the importance of OHSM in promoting healthy work environments. While OHSM is recognized as a critical component of organizational management, its integration into daily practices, parti­cularly in the public sector, remains inadequate. A notable contribution to Swedish research is the work of Rydell et al.,^18^ who emphasize the Plan-Do-Check-Act (PDCA) model as a fundamental framework for continuous workplace improve­ments, consistent with national and inter­national regulations,^19,20^ and essential for ensuring a healthy work environ­ment. Research shows that OHSM offers substantial benefits across both private and public sectors, including improved employee well-being and increased produc­tivity.^5,10,11^ Nevertheless, in the public sector, OHSM is often treated as a peripheral concern rather than an integrated part of manage­ment practices.^21,22^

Further research is also needed to examine the relationship between OHSM and digitali­zation.^1^ This study aims to address this knowledge gap by exploring how Swedish local government managers handle digitalization pro­cesses in relation to OHSM, and by identifying the potential drivers and barriers that influence their integration. Three research questions guide the analysis: 1) *What are the driving forces for managers to integrate digitalization in OHSM? 2) What are the barriers that distract managers from integrating digitalization in OHSM? 3) At which stages of the PDCA model are these drivers and barriers most prominent?*

The study draws on semi-structured interviews with managers, HR professionals, digitalization strategists, and Health and Safety representatives (HSRs) in two Swedish local governments. By applying the PDCA model, this study advances the theoretical understanding of OHSM by underlining how managerial approaches and organizational context shape the integration of digitalization with OHSM. The findings underscore the need for structured and reflective managerial practices to be able to succeed in this endeavor.

## Background

### Previous research on Occupational Health and Safety Management

Legal requirements and certification standards have fostered a more systematic approach to managing occupational health and safety (OHS) risks.^23^ For example, ISO 45001:18 is a global standard, aimed at reducing work-related harm and promoting well-being.^24^ It supports organizations in all sectors in developing preventive systems and aligns with other ISO standards. The standard requires employers to integrate OHSM into strategic decision-making as they shape the working conditions that could lead to OHS risks.^11,14,24^ However, the nature and extent of this responsibility varies globally.^6^ In some countries, OHSM is mandatory and regulated by law. In others, it relies on voluntary initiatives, such as policy development, risk assessment, and risk management,^24^ often guided by international, national or regional standards and guidelines.^15,22^

Sweden introduced OHSM regulations in the 1970s and was among the first European nations to adopt the EU Health and Safety Directive 89/391/EEC.^6^ Between 1996 and 2000, growing global interest in OHSM prompted the Inter­national Labour Organi­zation (ILO) to establish a comprehensive OSHM system (OHSMS) framework in 2001.^15^ OHSMS encompasses a broad spectrum of practices,^11^ ranging from proactive efforts to eliminate work place health risks to organizational strategies aimed to control employees and weaken unions. Such tactics are often associated with poor OHS outcomes (ibid). Despite its widespread use, the concept of OHSMS lacks a universally accepted definition.^12^ The ILO defines it as a “set of inter­related or interacting elements to establish OHS policy and objec­tives, and to achieve those objectives” [,^35^ p.10].

The rise in psychosocial ill health has prompted the expansion of OHSM to include psycho­social risks, now recognized as a top priority for occupational safety and health (OSH) in Europe.^2^ According to the EU Directive 89/391/EEC, such risks encompass aspects of work design, organizational structure, management practices, and social dynamics that may poten­tially harm employees’ health and well-being.^2^ Although the directive requires employers to assess and address all risks to employees’ health and safety, its references to psycho­social risks and work-related stress remains vague.^25^

### Drivers for Occupational Health and Safety Management systems

Drivers for OHSMS can be grouped into three categories: organizational com­mit­­ment, organi­zational synergies, and OSHM practices.^12^ *Organizational commitment* encompasses employee awareness, employee participation and management commitment and leadership. *Organi­zational synergies* include Trade Union involvement, participatory and flatter organi­zational structures and cooperative relations within labor-markets. *OHSM practices* include training, risk assessment, clearly defined responsibilities, effective communication and disse­mination of results, OHS policies and programs, supervision, and safety work procedures. An OHSMS depends on ongoing efforts to refine and develop its constituent processes. This requires strong managerial and senior leadership support, the develop­ment of proactive OHSM, improved allocation of financial resources, and a commitment to continuous improvement in OHS processes.^24^ As these systems typically follow a cyclical model, where steps are repeated regularly, continuous improvement is essential to maintain their relevance and effec­tive­ness.

Beyond safeguarding employees’ well-being through targeted activities and initiatives,^12,13^ a key driver for OHSM is the reduction of costs associated with work-related injuries and illnesses, including medical expenses and sick leave: costs that can negatively impact produc­tivity.^26^ Given the centrality of increased productivity and cost efficiency to the strategic vision of the Swedish public sector,^4^ a sociotechnical argument for investing in healthy work­places is not to view it merely as expenditure, but rather as a strategic contri­bution to long-term sustainability through enhanced employee health and well-being.^27^

### Barriers to Occupational Health and Safety Management systems

Barriers to OHSMS can be grouped into the three main categories^12^: organizational commit­ment; availability of resources; and OHSMS practices. *Organizational commitment* refers to challenges such as lack of manage­ment commitment, lack of knowledge regarding the impor­tance of OHSM, differing visions between managers and OHSM objectives, low levels of employee participation in decision-making, and a weak safety culture. *Availability of resources* includes the lack of specialized personnel, financial constraints and time limitations. Barriers to *OHSMS practices* includes the frequent underestimation of risks within organi­zations and inadequate communication. Under­estimating OHS risks in the context of digitali­zation may reflect a techno­logical deterministic mindset, characterized by an uncritical belief in the inherent value of technology as a resource,^28–31^ which is often assumed to enhance the creation of meaningful work processes.^32^ Da Silva and Amaral^24^ identify further barriers, including high implementation and manage­ment costs, difficulties in integrating various policies and aligning them with organizational culture, failures in risk assessment processes and challenges in maintaining effective OHS control and documen­tation systems. Moreover, Frick^32^ emphasizes that the lack of inter­action between managers, employees and their safety representatives, constitutes a significant barrier to achieving an effective OHSMS. Frick and Kempa^11^ further suggest that although worker consultation is frequently emphasized in OHSM guidelines, it often functions more as a mechanism for compliance with managerial directives than as a platform for meaningful dialogue about OHS goals and strategies.

These drivers and barriers underscore the critical role of the managerial approach in shaping OHSMS outcomes. Strong management commitment is consistently identified as essential for the successful implementation of OSHMS, while its absence constitutes a major barrier to OHSMS development.^12^ However, the complexity of changing organizational policies suggests that effective OHSM integration requires both managerial commitment and active support from senior leadership.^24^

### Digital governance and Occupational Health and Safety Management

Political pressure and the implementation of New Public Management (NPM) principles have brought about substantial changes in the public sector, both in Sweden and other countries.^33,34^ A prominent outcome of decades of NPM-inspired reforms, prioritizing efficiency and cost reduction with digitalization promoted as a central solution,^35,36^ is the emergence of digital governance, aimed at enhancing efficiency, reducing expenditure, and increasing trans­parency. An example of limitations with digital governance is that it can reinforce silo thinking, which implies an organizational tendency to resist cross-functional collaboration, which in turn risks hindering integrated approaches to complex challenges.^33^

While the pursuit of productivity and efficiency through digitalization is not inherently at odds with the implemen­tation or maintenance of OHSM,^17^ these structural and cultural shifts have had considerable implications for both working conditions and the management of OHS.^33^ For example, digital technology that fails to provide sufficient support or resources to employees may contribute to adverse psychosocial outcomes, such as reduced well-being.^37^ These effects can diminish productivity and efficiency, ultimately undermining organi­zational performance and financial sustainability.^16^

### The Plan-Do-Check-Act model for compliance with OHSM

The Plan-Do-Check-Act (PDCA) model (figure 1), developed by Edward Deming, is a flexible and structured tool that can be used for planning, implementing, and improving OHS initiatives.^38^ It outlines four iterative steps designed to drive continuous improvement in organizational processes^39,40^: Plan (identify a change aimed at improve­ment); Do (implement and test the change); Check (analyze the results); and Act (make necessary adjustments and plan for the next cycle).

**Figure 1.**
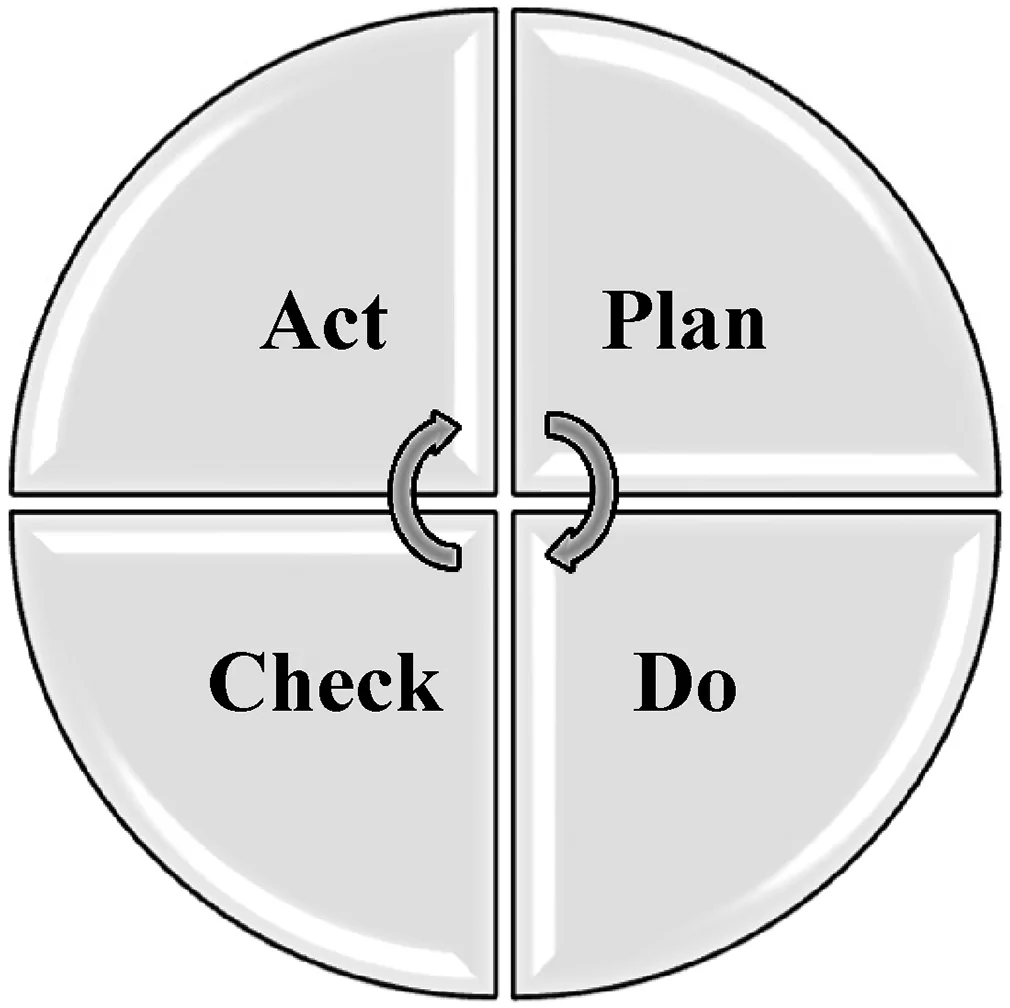
Plan-Do-Check-Act model, inspired by Taylor et al. [40].

The iterative cycle of the PDCA model is well-suited for complex systems,^40^ such as those involving the interaction between individuals, procedures and equipment when working collectively toward a common goal.^39^ Since 2018, the PDCA model has been incorporated into the inter­national standard ISO 45001 for OHSM, which provides a structured framework for managing occupational risks and promoting both physical and psychosocial health.^41^ Moreover, the model aligns with international work­place safety legislation, including Directive 89/391/EEC, and is widely used as a guiding principle to support comp­liance with OHSM regulations and recommendations.^19,20^ Organizations such as the ILO,^14^ the European Agency for Safety and Health at Work,^42^ and the Swedish Association of Local Autho­rities and Regions^43^ have all based their OHSM guidelines on the PDCA framework.

### Occupational Health and Safety Management in Sweden

Swedish OHSM is historically rooted in the relationship between local trade unions and health and safety representatives.^44^ Certain OHS matters are regulated through collective agreements between trade unions and employers, tailored to specific organizational and workplace conditions.^45^ However, since 1977, all Swedish workplaces have been legally required by the Swedish Work Environment Act to establish an OHSM system to support employee health and well-being.^19^

Psycho­social risks, including work-related stress, are explicitly addressed in the Swedish national legislation.^46^ One of the binding provisions is the Systematic Work Environment Management (SWEM), introduced in 2001 (referred to in Swedish as SAM).^21,33^ SWEM mandates that employers systematically investigate, monitor, and take preventive action to reduce the risk of accidents and illness among employees.^47^ SWEM is further described as a collaborative process in which management and employees (or their represen­tatives) jointly “examine the work environment; investigate incidents and accidents; conduct risk assessments; develop action plans and implement improve­ment measures, and evaluate the results in terms of risk elimination” [,^20^ p. 108].

SWEM encompasses both the organizational and the social work environment. The organi­zational dimension, which refers to management and governance, participation, assignment of tasks and requirements, resources and respon­sibilities is increasingly transformed by digital technology in the form of algorithmic management.^48^ The social dimension, which refers to colla­bora­­tion, managerial support and social interactions,^49^ increasingly takes place through digital channels such as email, text messages and social media. Resources for creating a healthy work environment referred to in the law include work processes and equip­ment, skills and staffing, reasonable and clear goals and opportunities for control at work.^49^

In the Swedish public sector, politically appointed boards with OHS responsibilities often delegate OHSM duties to senior management,^45^ who in turn pass these responsibilities onto line managers.^44^ However, this delegated respon­sibility is rarely integrated into day-to-day operational management, often being treated as a “side wagon”.^22,33^ Moreover, managers do not consistently integrate digitali­zation into OHSM practices.^10^

Despite the existence of formal OHSM strategies within Swedish public sector organizations, studies have identified inadequate interaction between manage­ment and safety committees.^33^ Addi­tionally, OHS related communication from mana­ge­­ment to employees is frequently limited.^33^ Both of these shortcomings are problematic, as they represent key elements of an effective OHSM for achieving a healthy work environ­ment.^18^

## Methods

One urban and one rural municipality participated in the study. In Sweden, a municipality is classified as urban if the largest urban area has at least 200,000 inhabitants, while a rural municipality has less than 15,000 inhabitants in its largest urban area.^50^ Although Swedish municipalities vary in population size, they all share the same responsibility to provide welfare services in accordance with national standards.^51^

### Data collection

Two units from each municipality were selected for inclusion in the study. The selection was made by the research team in dialogue with top managers and strategists within the respective organizations.

A total of 29 semi-structured interviews were conducted with managers, digitalization strate­gists, HR professionals and Health and Safety Represen­tatives (HSR) (see Table 1). HSRs were included due to their role in representing employees in OHSM matters.^11^ These interviews provided in-depth under­stan­ding of how OHSM considerations were addressed during the implementation of digital technology. The diverse roles of the respondents contributed to a broad understanding of how OHSM was integrated into digitalization processes. To ensure con­­fiden­­­tiality, all partici­pants were anony­mized. Prior to participation, individuals received both verbal and written information about the study and signed a written consent form.

**Table 1.** Overview of respondents and digital technology in the two participating local governments.

**Urban Local Government (ULG)**	**Respondents**	**Digital technology**	**Qualitative approach**	**When**	**How**
Managers	3 top-level managers 2 specialists/strategists 3 Health and Safety representatives		Semi-structured in-depth interviews	March – Dec 2020	2 face-to-face 1 phone 11 video conferences
School managers	2 top-level managers 1 specialist/strategist	Systems for admin. and education, e.g., School­soft, Teams, Digi-Exam, Classroom			
Social services managers	2 top-level managers 1 specialist/strategist	A software robot with a decision-making intelligence			

**Rural Local Government (RLG)**	**Respondents**	**Digital technology**	**Qualitative approach**	**When**	**How**
Managers	3 top-level managers 2 specialists/strategists 3 Health and Safety representatives		Semi-structured in-depth interviews	March - July 2020 September 2021	Video conferences
School managers	2 top-level managers 1 specialist/strategist	Systems for admin. and education, e.g., ItsLearning, Endurance, Teams, Dexter.			
Home Care service managers	1 top-level manager 3 front-line managers	App for home care service. LifeCare, Procapita			

The interviews were conducted in Swedish and transcribed verbatim. The quotations included in this paper remain as close to the original transcrip­tions as possible, though minor adjustments were made to enhance clarity when translating from Swedish to English and to ensure co­herence outside their original context.

To provide contextual background, we reviewed digitalization strategies, annual reports, and employee surveys from both municipalities. Additionally, to better understand the visions and goals guiding the managers’ digitalization efforts, we examined e-government reports and digitali­zation strate­gies from national organizations, such as the Swedish Association of Local Authorities and Regions, as well as from international bodies including the Organisation for Economic Co-operation and Development and the United Nations.

### Analysis and structuring of data

The analysis followed an inductive coding approach inspired by Gioia et al.^52^ This involved reading and interpreting the raw textual data to identify emerging concepts and themes. The first author began by organizing prominent narratives from the interview transcripts into *first order-concepts.*^53^ These were derived from close readings of textual units (e.g., words, sentences or paragraphs), while carefully preserving the terminology used by the informants. To maintain confidentiality, all data were coded. Similar to Gioiás methodology, the initial coding process generated a plethora of categories,^52^ which were then discussed and refined colla­bora­tively by all three authors. Examples of salient first-order concepts include: *managers collaborate with HR during digitalization, but neglect to address the work environ­ment*; *digitali­zation and reorganization occur simultaneously without risk assessment*; and *HSRs are involved in all OHSM matters except digitalization*.

In the second stage, each author examined whether the first-order concepts could be merged into second-order themes that capture underlying patterns and are supported by theory. Examples of second-order themes include: *Digitalization oriented focus, Technological deter­minism* and *Limited key stakeholder involve­ment*. These were then further abstracted into the two aggre­gated dimen­sions: *Potential drivers for integrating digitalization and OHSM* and *Potential barriers for integrating digitalization and OHSM.*

The coding process was iterative and continued until a consensus was reached on the data's structure and meaning. The final structure, illustrating the progression from raw data to aggre­gated dimensions, is presented in Figure 2.

**Figure 2.**
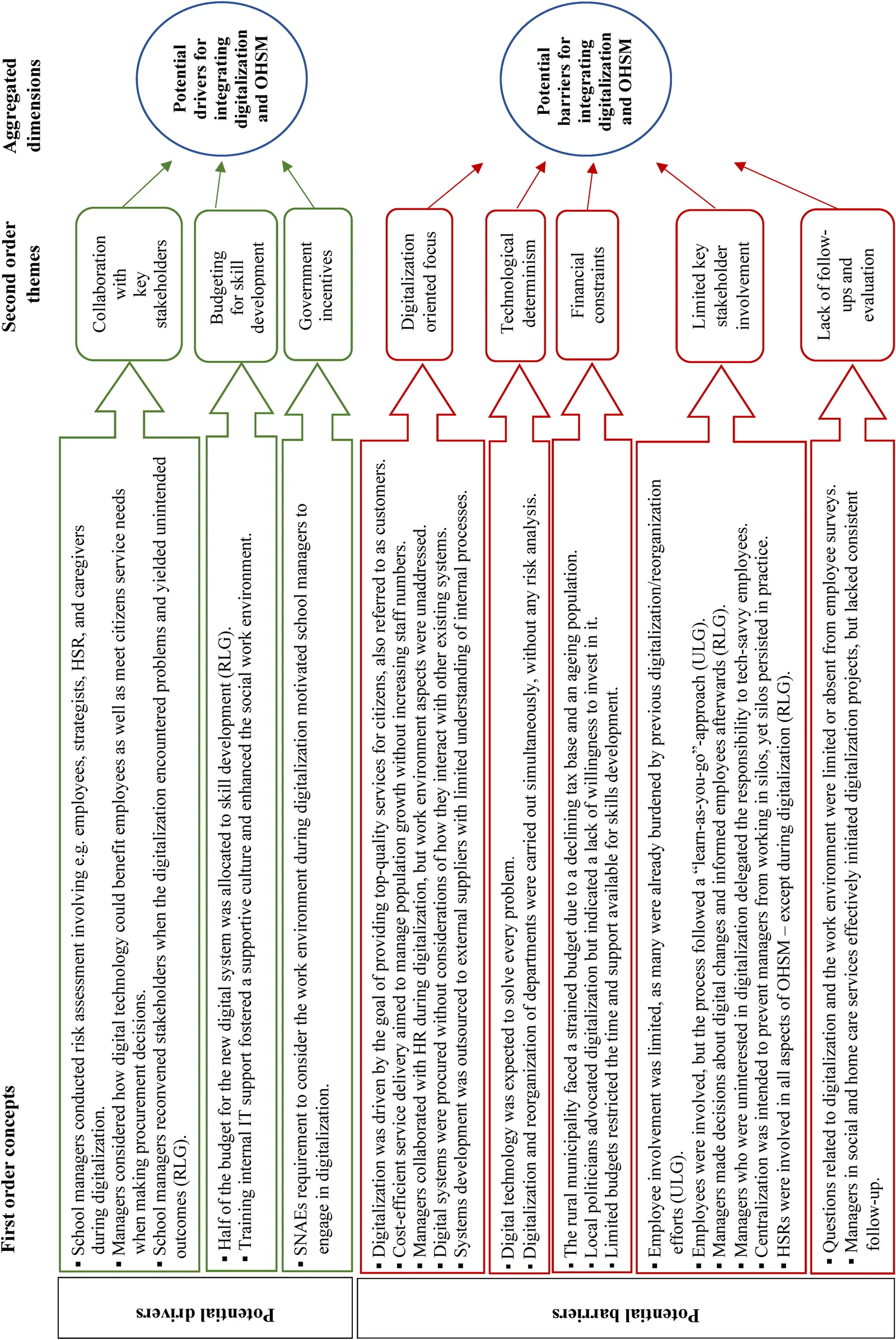
Potential drivers and barriers for integrating digitalization and OHSM.

In the third stage, to theoretically anchor the empirical findings,^52^ these were linked to the PDCA model. The model was chosen for its flexible and iterative structure, and its incorpo­ration into the revised framework of the ISO 45001:2018 standard.^23^ This analytical approach facilitated the identification of phases in which the most salient potential drivers and barriers emerged. An analysis of the second-order themes in relation to PDCA, indicated for example that *collaboration with stakeholders* and *lack of follow-ups and evaluation*, appeared across multiple PDCA phases. To further refine the understanding of specific drivers and barriers shaping the integration of digitalization and OHSM, we revisited the first-order concepts and positioned them onto the PDCA model (see Table 2).

**Table 2.** Illustration of how second order themes and first-order concepts were positioned to the PDCA model. The first five to eight words of the first-order concepts are presented in the table, while the full text is found in Figure 2.

PDCA phase	Core theoretical concept	Potential drivers and barriers for integrating digitalization and OHSM (Second order themes)	Managers’ enactment of digitalization and OHSM (First order concepts)
Plan	Identification and planning of changes for improvement	*Drivers:* Collaboration with key stakeholders	School managers conducted risk assessment involving e.g., employees…
		Budget for skill development	Managers considered how digital technology could benefit employees… Half of the budget for the new digital…
		Government incentive	SNAEs requirement to consider the work environment during…
		*Barriers:* Digitalization oriented focus	Digitalization was driven by the goal of providing… Cost-efficient services delivery aimed to manage population growth… Digital systems were procured without considerations of how…
		Technological determinism	Digital technology was expected to solve all problems.
		Financial constraints	The rural municipality faced a strained budget… Local politicians advocated digitalization but indicated a lack of…
Do	Implementation and testing of planned changes	*Drivers:* Collaboration with key stakeholders	School managers collaborated with HSRs in OHSM meetings.
		Budget for skill development	Training internal IT support fostered a supportive culture…
		*Barriers:* Digitalization oriented focus	Managers collaborated with HR during digitalization…
		Technological determinism	Systems development was outsourced to external… Digitalization and reorganization of departments…
		Limited stakeholder involvement	Employee involvement was limited, as many… Employees were involved, but the process… Managers made decisions about digital changes… Managers who were uninterested in digitalization… Centralization was intended to prevent managers… HSRs were involved in all aspects of OHSM-except…
Check	Monitoring and analysis of work environ­ment outcomes	*Drivers:* Collaboration with key stakeholders	School managers reconvened stakeholders when the digitalization encountered…
		*Barriers:* Limited stakeholder involvement	HSRs were involved in all aspects of OHSM except during digitalization.
		Lack of follow-up and evaluation	Questions related to digitalization and the work environment…
Act	Adjustments and planning the next cycle based on gained insights	*Drivers:* Collaboration with key stakeholders	School managers collaborated with HSŔs in OHSM meetings when digitalizing.
		*Barriers:* Lack of follow-up and evaluation	Managers in social and home care services effectively…

## Results

This section begins with the potential drivers, organized according to our second-order themes: *colla­bo­ration with key stake­holders; bud­geting for skill development; government incentives to promote digitalization in OHSM at local government level.* Thereafter, the potential barriers, also organized according to our second-order themes are presented: a *digitali­zation-oriented focus; technological determinism; financial const­raints; limited key stake­holder involvement; lack of follow-up and evaluation mechanisms*. Finally, a brief description is provided of the stages of the PDCA model in which these drivers and barriers are most prominent.

### Potential drivers for integrating digitalization and OSHM

#### Collaboration with key stakeholders

In the context of school management, both managers and strategists actively involved stake­holders, such as caretakers, pupils, employees and HSRs, during the planning and procurement of a new digital system. The digitalization of school operations was seen both as a means to benefit pupils and/or parents and also to improve working conditions for employees.

One top school manager emphasized the importance of avoiding top-down system design, advo­cating instead for inclusive approaches that incorporate input from all relevant parties. A top manager from the Urban Local Government (ULG) reflected on the importance of inte­grating digitalization into OHSM:

That is something we should always do, actually - include those who will be using the systems and not cut corners. I feel that sometimes we might rush through this process. We may not have the time, but it is important. It costs money and effort to learn something new, and it needs to work well for a long time. So, I do not think it is good to be careless in this regard.

These managers demonstrated a clear understanding of the importance of integrating digital technology into OHSM, particularly in terms of how systems and standards affect employee health, skill development and engagement.

In the Rural Local Government (RLG) school setting, managers collaborated with key stake­holders, including HSRs, to conduct follow-ups after implementing new digital techno­logy. In this process, problematic aspects of the new digital technology were identified, which enabled early decisions to be made regarding necessary corrections or adjustments.

#### Budgeting for skill development

In the RLG school context, the procurement of a new system included a pre-allocated budget, with half of the funds dedicated to employee training from the outset. This budget supported the hiring of external consultants to train key personnel. However, managers recognized that training internal staff was more cost-effective in the long term, as consultants were considered “extremely expensive”.

Moreover, training internal personnel fostered peer support and collaboration. A top-manager noted:

If I know the person who is going to help me, it usually becomes much easier to ask for help. I can actually be a bit blunt with my colleague, so to speak. ‘You know, I don't understand this.’ I mean, you can say that. And it can even be like, ‘Hey, I know you have some trouble with this. Should I sit down with you this afternoon and we can do this together?’ A consultant doesn't do it in the same way. So, I think the personal aspect is quite important there as well.

#### Government incentives to promote digitalization in OHSM at local government level

Managers in both the urban and rural school settings tended to be more proactive in integrating digitalization with OHSM compared to those in social or home care services. A senior manager in RLG described the impact of an initiative to promote digitalization in schools by the Swedish National Agency for Education (SNAE)^54^:

It motivated us to accelerate our efforts. The education sector took the lead, helping to guide other local government services, much like a train pulling carriages. We had specific obligations to fulfill, leaving us no choice but to comply.

Although digitalization received increased attention, managers continued to consider its impli­cation for the work environment. They involved employees and HSR to mitigate potential OHSM risks and allocated funds for training during system procurement.

The SNAE report not only encouraged digitalization but also emphasized the importance of considering its impact on the work environment, referencing relevant research at the inter­section between digitalization and occupational health.

### Potential barriers for integrating digitalization and OHSM

#### Digitalization-oriented focus

According to policy documents and interviews, a key objective of digital technology imple­men­tation was to streamline public services and align them with citizens’ needs. Both municipalities aimed to enhance efficiency and service quality. In the ULG, the board of directors emphasized accelerating digital trans­for­­mation to achieve service excellence and cost-effectiveness. Work processes were stream­lined to maintain productivity amid population growth without increasing staff. In contrast, RLG managers reported limited strategic direction and support from the local government council in guiding their digitalization efforts, viewing digital technology mainly as tools for citizen inter­action. Nevertheless, managers in both munici­palities aimed to promote efficiency, transparency and accoun­tability through digitali­zation, for example, by facilitating processes involving assessment.

Digitalization was also viewed as a strategy to enhance employer attracti­veness. Managers emphasized benefits such as reducing repetitive tasks and long-distance travel, enabling employees to focus on more meaningful work. However, despite this employee-centered perspective, managerial discussions often centered on digitalization itself. As one RLG manager noted, “Conversations often focused on robots, AI, software, and machines. In all this talk, the core idea of digitali­zation changing the way we work, seems to have been somewhat overlooked”.

In the ULG, a manager noted that although HR was involved in digitalization of administrative services, its potential impact on the work environment was overlooked. Furthermore, HSRs participated only in formal negotiations and not in risk or consequence analyzes to related work process changes.

Overall, managers in both municipalities appeared to prioritize a citizen-centric and economic focus over a healthy work environment when digitalizing work processes. As a top manager in the ULG explained, the goal was not solely on financial efficiency, but also effective service delivery. Employees were expected to under­stand their role in the public service and the importance of using tax funds responsibly, to ensure appropriate resource allocation.

A recurring challenge identified in the study was the lack of interoperability between digital technologies. A top manager in the ULG described the impact of digitalization on the employees’ work:

There are horror stories within our organization where employees in certain departments work with between 10 and 20 systems. This is because we have picked items [digital technology] off the shelf, thinking, “Well, this one fits here, and that one fits there,” instead of considering, ‘What do we need for process A to Z to function well?’ […] We should aim to purchase something that we can adjust and refine, rather than something that is very rigid.

Another issue raised by the managers was the outsourcing of IT development to external pro­viders. One RLG manager described this as being at the mercy of IT developers, given their limited influence over their work and little understanding among developers of how new systems might affect the work environ­­ment.

#### Technological determinism

Overconfidence in the technology was evident in several interviews. A middle manager in the ULG, described a robot by stating, “never making mistakes […], never taking care of sick children, working 24/7, and being so good”, implying that full digitalization would inherently ensure optimal productivity outcomes. A top manager criticized this opti­mistic attitude, noting that some managers pursued digitalization merely “for the sake of digitali­­zation”.

A reorganization in the ULG, coinciding with the automation of certain work processes, led to considerable employee dissatisfaction. Many perceived the changes as being driven by an overconfidence in technology. Consequently, several employees resigned, and their positions were not immediately filled, resulting in a reduced workforce and increased work­load for those who remained.

A HSR highlighted the risks associated with techno­logical determinism:

You cannot start downsizing people before we have achieved this fantastic digitalization that is supposed to solve everything. I have this robot [name of the robot]. It sounds great, but the robot also makes a lot of mistakes, although no one says that publicly.

Unanticipated shortcomings in the automated process further intensified the workload for the remaining staff, underlining a disconnect between technological expectations and operational realities.

#### Financial constraints

In both the RLG and the ULG, a declining tax base, primarily due to ageing populations, was cited as a key reason for identifying digitalization as a strategic solution. The interviewed managers expressed a shared objective: to achieve a sustainable welfare economy while main­taining budgetary balance, in alignment with the goals set by the Swedish government.^4^

The following quote from a top manager in the RLG illustrates the financial strain and the perceived lack of engagement and strategic direction from the local government council regarding digitalization:

They [politicians] often want us to be on the frontline and to be very digital and everything. But the cost involved is another matter; they usually do not consider that it (digitalization) just has to be done, and money has to be saved. We are supposed to reduce employees because we are going digital, which means that a lot of tasks disappear, and they will. But it also leads to many other things that they do not think about. […].

Given the economic constraints and limited political interest, RLG managers focused on opti­mizing and utilizing existing systems, rather than investing in new technologies.

Financial constraints and the pursuit of cost-efficiency also resulted in reduced training oppor­tunities for employees in both local governments. Employees were often expected to develop digital skills during their spare time, a resource that was frequently unavailable. A HSR in the school setting reflected on this issue, “Have you heard of many teachers that have spare time? […] as long as it is voluntarily, it [self-initiated training for digital skills] will not happen”.

#### Limited key stakeholder involvement

An example of limiting stakeholder involvement observed in the ULG, was social service managers choosing not to involve employees in the digitalization process. Their rationale was that employees were already experiencing a high workload, and additional responsibilities might overburden them. In retrospect, one ULG manager reflected, “If I could do it again, I would involve the employees much, much earlier, because I believe it always turns out better that way”.

Another manager initially assumed that digitalization would bring immediate benefits to employees, but later came to recognize the importance of early involvement, rather than relying on a “learn-as-you-go” approach.

In the RLG, stakeholder involvement was limited in a different way. Managers selectively engaged employees who were technically skilled and positively inclined toward digita­lization, thereby risking the exclusion of perspectives from those less technically skilled. In the home care sector, the lack of inclusive involvement was evident as employees were only engaged after the decision to implement digital technology had already been made.

The study also identified limited involvement of HSRs during the digitalization process. For instance, within the ULG, managers and HR departments excluded HSRs from discussions con­cerning digitalization, despite efforts to centralize decision-making within a dedicated digi­tali­­zation unit aimed at preventing fragmented solutions across departments. These findings in­di­cate that organizational silos continue to exist in practice, particularly within social services, concerning the integration of digitalization into OHSM.

#### Lack of follow-up and evaluation

Managers in both social and home care services lacked systematic follow-up procedures to evaluate the impact of digitalization on the work environment. This absence of structured assessment limited their understanding of whether, and in which ways, employees had been affected by the digitalization of work processes. One top manager in the RLG reflected on this challenge:

The big challenge is always to really check everything [laughs]. If everything works according to the plan, we are not always thorough about following up on every single point, as long as we know it has worked. It is when things do not work that we really dive in and look closely. So, it is definitely an area for improvement.

In addition, employee surveys did not include questions related to the effects of digital technology on the work environ­ment, nor the availability of social support from managers and colleagues. This omission further limited insight into how digitalization influenced employee well-being and organizational dynamics.

### Potential drivers and barriers throughout the PDCA model

Collaboration with key stakeholders emerged as a potential driver across all four PDCA phases, while budgeting for skill development appears in the Plan and Do phases, and government incentives are primarily evident as drivers in the Plan phase. However, technological deter­minism and financial constraints constitute potential barriers in the Plan phase, whereas a digitalization-oriented focus is evident as a potential barrier in both the Plan and Do phases. Limited stakeholder involvement appears as a barrier in the Plan, Do and Check phases, and a lack of follow-up and evaluation are observed as key barriers in the Check and Act phases. That the potential barriers overshadow the potential drivers, indicates that attention to OHSM concerns is diminished, which may constrain the integration of digitalization into OHSM and limit opportunities for continuous improvement within a digital work setting. The implications of applying our second-order themes and first-order concepts to the PDCA model for under­standing the integration of digitalization and OHSM are further addressed in the discussion section.

## Discussion

This study explored how Swedish local government managers handled digitalization, in relation to OHSM, identifying potential drivers and barriers for integrating these two areas of responsibility. The study was guided by the following research questions:

1) What are the driving forces for managers to integrate digitalization in OHSM? 2) What are the barriers that distract managers from integrating digitalization in OHSM? 3) At which stages of the PDCA model are these drivers and barriers most prominent?

Earlier research has shown that the success of OHSM depends on a range of factors that can act as either drivers or barriers.^11,12,18,24^ This study identified three potential drivers and five potential barriers associated with the integration of digitalization into OHSM within local government contexts. At first glance, the drivers appeared to outweigh the barriers, as all participating managers expressed support for OHSM, while simulta­neously embracing digitalization. However, the managers adopted two distinct approaches: one that seemed to facilitate the integration of digitali­zation into OHSM and another that did not. The following section focuses on the key drivers and barriers and outlines where in the digitalization process they were found, helping explain these divergent approaches.

### Key drivers behind managerial integration of digitalization in the OHSM

School managers appeared to adopt a systematic approach aligned with the PDCA model,^40,55^ aiming to minimize OHS risks from the initial procurement phase through to post- implementation follow-up. These managers demonstrated what has been referred to as procure­ment competence,^56^ enabling them to consider employees’ OHS needs already during the procurement stage. Furthermore, they collaborated with employees, HSR and external stakeholders, such as care givers and pupils, to conduct appropriate risk assessments during the digitalization process.

Vitrano and Micheli^12^ have emphasized management commitment, risk assessment, employee partici­pation and trade union involvement as key drivers of successful OHSM systems**.** An additional driver identified in this study is the influence of higher-level decision makers who encourage managers to consistently consider work environment issues, as previously highlighted by Lornudd et al.^57^ Similarly, Da Silva and Amaral^24^ underscore the importance of senior management support for effective OHSM implementation.

In this study, such support was exemplified by the encouragement from the Swedish National Agency for Education (SNAE), which may have influenced school managers to adopt a proactive approach to integrating digitalization into OHSM. Despite financial constraints, commonly identified as a barrier to OHSM,^12^ school managers in the RLG demonstrated the ability to integrate digitalization in OHSM. By strategically allocating resources^24^ and involving key stakeholders, they fostered a comprehensive understanding of the intercon­nection between digitalization and employee well-being^37^ throughout the digitalization process.

In summary, school managers seemed to employ a systematic approach to minimize OHS risks, demon­strating procurement competence and engaging key stakeholders throughout the pro­cess. Effective drivers included management commitment, employee and HSR partici­pation and support from higher-level authorities. Even under financial constraints, managers succee­ded in integrating digitalization into OHSM, supported by institutional encouragement from the Swedish National Agency for Education (SNAE).

### Key barriers that distract managers from integrating digitalization in the OHSM

Managers in social and home care services tended to adopt an ad hoc approach to digitalization, rather than working systematically with risk assessments early in the process and evaluations of its effects later in the process. This approach reflected a predominantly positive perception of digital technology, which tended to limit their consideration of its potential psychosocial impacts on the work environment.^6^ Such an approach can be described as a form of technological determinism,^58,59^ where the potential OHS risks associated with digitali­zation are underestimated, an issue previously identified as a significant barrier to effective OHSM.^12^

Technological determinism may also have influenced managers’ understanding of the impor­tance of involving employees or HSR in OHSM. For example, while social and home care managers involved HR and HSR in risk assessments for most work-related processes, they excluded digitalization-related changes from this practice. This tendency to treat OHSM as a secondary concern, or as a “side wagon”, as described by Håkansta et al.,^59^ further illustrates the influence of technological determinism.

Another prominent barrier was observed during the implementation of automated processes in social services, which coincided with a reorgani­zation that divided one work group into two separate units. Managerial decisions during these processes indicated a lack of commitment, insufficient risk assessments, and lack of follow-up, elements previously identified as barriers to effective OHSM systems.^12^ Our findings suggest that such barriers are compounded by limited collaboration with health and safety committees and poor communication regarding OHSM, aligning with Frick.^32^

Moreover, there were notable shortcomings in fostering meaningful dialogue and establishing clear OHS objectives and strategies,^11^ particularly in social and home care services in relation to digitalization. This managerial approach contrasts with Demerouti's recommen­dation that managers should ensure that “job resources are maximized and job demands are manageable” in order to promote meaningful work, particularly during digitali­zation processes [,^36^ p.1205]. When these aspects are overlooked, it may indicate that risks are being under­estimated within the organization.^12^

Although managers in social and home care services expressed good intentions regarding digitalization and its potential to improve the work environment, these intentions were often overshadowed by various barriers, especially during the Plan phase (table 2). This pattern appeared to persist throughout the entire digitalization process. Had these managers adopted an iterative cycle, such as the Plan-Do-Check-Act (PDCA) model,^39,40^ it could have provided a structured framework for continuously identifying and managing risks.^23^

By highlighting the importance of managerial approach and organizational context in shaping the integration of occupational health and safety within digitalization processes we contribute to the theoretical understanding of OHSM. By contrasting systematic and ad hoc approaches, the findings underscore the value of applying iterative cycles, particularly the PDCA model, as a conceptual framework for managing OHS risks in dynamic and technology-driven environ­ments. The study extends the application of PDCA beyond traditional industrial settings, demon­strating its relevance in public sector domains such as education and social care. Furthermore, the identification of technological determinism as a barrier enriches the conceptual discourse on OHSM by introducing a critical lens through which managerial assump­tions about digitalization can be examined. These insights collectively advance the conceptualization of OHSM as a dynamic, context-sensitive process that benefits from structured methodologies and reflective managerial practices. This study also offers important implications for occupational health and safety research by demonstrating how digitalization processes intersect with managerial practices and organizational structures in shaping OHS outcomes.

### Methodological considerations

A strength of this study lies in the breadth and diversity of its empirical material. It draws on 29 semi-structured interviews across four organizational units in two municipalities, involving managers, strategists, HR professionals, and HSR's. This diversity provided rich insights into how digitalization and OHSM were understood and managed at different organizational levels. Triangulation through municipal policy documents, national strategies, and agency reports further strengthened the validity of the analysis.

Using the Gioia methodology enhanced analytical rigor by systematically moving from first-order concepts, to second-order themes and aggregated dimensions. This iterative process reduced researcher bias and ensured transparency in developing theoretical interpretations. Mapping the second-order themes as well as the first-order concepts onto the PDCA model added analytical depth and provided a structured way to interpret drivers and barriers of digitalization within OHSM.

Nevertheless, some limitations remain. Focusing on two municipalities limits generalizability, and differences in size and resource may have influenced managerial approaches. The interviews were conducted during the COVID-19 pandemic, which may have affected both managerial practices and participants’ reflections. Moreover, while the Gioia approach promotes transparency, categorization and abstraction involve interpretive choices, and alternative coding could yield different emphases.

Finally, the study lacked systematic observations of digitalization in practice and a comparative perspective beyond Sweden. Future research could employ longitudinal or ethnographic designs to explore how drivers and barriers evolve over time and how managers navigate the PDCA phases in real-world contexts. A comparative perspective would further deepen under­standing of these dynamics across institutional and international contexts.

## Conclusion

This study highlights the complex interplay between managerial practices, organizational context, and digitalization in shaping the integration of Occupational Health and Safety Management (OHSM) within local government settings. By identifying three potential drivers and five potential barriers, the findings underline how systematic approaches, particularly those aligned with the PDCA model, can facilitate proactive and inclusive OHSM integration, while ad hoc strategies risk overlooking critical psychosocial dimensions. The study advances conceptual understanding by extending the relevance of PDCA to public sector digitalization and by introducing technological determinism as a barrier to effective OHSM. These insights underscore the need for reflective, structured, and participatory management practices to ensure that digital transformation contributes to sustainable and health-promoting work environments. In doing so, the study contributes to occupational health and safety research by emphasizing the importance of context-sensitive frameworks and institutional support in navigating the challenges of digital change.
